# Dense hyperthermic intraperitoneal chemotherapy with cisplatin in patients with stage III serous epithelial ovarian cancer: a retrospective study

**DOI:** 10.1186/s12885-021-08507-y

**Published:** 2021-06-27

**Authors:** Xiaoli He, Li Wei, Rui Li, Shuang Jing, Linlin Jia, Danwei Ji, Yali Li, Yue Wang, Yongxia Zhu

**Affiliations:** 1 Department of Gynecology, Henan Provincial People’s Hospital, People’s Hospital of Zhengzhou University, People’s Hospital of Henan University, No.7, Weiwu Road, Jinshui District, Zhengzhou, 450003 China; 2 Department of Gynecology and Obstetrics, Taikang Xian People’s Hospital, No. 469, Jianshe Road, Taikang county, Zhoukou, 461400 China

**Keywords:** Dense hyperthermic intraperitoneal chemotherapy, Serous epithelial ovarian cancer, Survival, Interval debulking surgery

## Abstract

**Background:**

To investigate the efficacy and safety of interval debulking surgery (IDS) combined with dense hyperthermic intraperitoneal chemotherapy (HIPEC) with cisplatin in Chinese patients with FIGO stage III serous epithelial ovarian cancer (EOC).

**Methods:**

This retrospective single-center study reviewed the demographic and clinical data of 197 patients with primary FIGO stage III serous EOC who were treated with IDS with (*n* = 121) or without (*n* = 76, control group) dense HIPEC between January 2012 and April 2017. The co-primary endpoints were progression-free survival (PFS) and overall survival (OS), and the secondary endpoint was the occurrence of adverse events.

**Results:**

The median PFS was 24 months in the IDS plus dense HIPEC group, whereas it was 19 months in the IDS alone group (hazard ratio [HR] 0.46, 95% confidence interval [CI]: 0.33–0.65, *p* = 0.000). The median OS in patients treated with IDS plus dense HIPEC (51 months) was significantly longer than that in patients treated with IDS alone (40 months, HR 0.52, 95% CI: 0.35–0.78, *p* = 0.001). The demographic and preoperative clinical characteristics of these two groups were comparable (*p* > 0.05). In the IDS alone group, no adverse events were recorded in 42 (55.3%) of the 76 patients, and 14 (18.4%) patients were reported to have grade III/IV adverse events. In the IDS plus dense HIPEC group, no adverse events were recorded in 55 (45.5%) of the 121 patients, and 23 (19.0%) patients were reported to have grade III/IV adverse events. No postoperative deaths occurred within 30 days in either group and neither did severe fatal complications in the IDS plus dense HIPEC group.

**Conclusions:**

IDS plus dense HIPEC with cisplatin in Chinese patients with FIGO stage III serous EOC is associated with improved survival and is reasonably well tolerated by patients.

## Background

Epithelial ovarian cancer (EOC) has the highest mortality rate among all gynecological tumors worldwide, causing nearly 22,500 deaths in China in 2015 and 14,335 deaths in the United States in 2018 [1, 2]. The most effective treatment for advanced EOC remains primary cytoreductive surgery (CRS) followed by intravenous chemotherapy with carboplatin and paclitaxel [3, 4]. In patients where complete primary cytoreduction is impossible, interval debulking surgery (IDS) can be performed after 2–4 cycles of neoadjuvant chemotherapy (NACT) [5–7]. Peritoneum is the primary site of metastasis of tumor cells from the ovary in advanced EOC patients [8, 9]. Therefore, intraperitoneal chemotherapy is used to improve outcomes and minimize tumor dissemination and implantation [10, 11].

Hyperthermic intraperitoneal chemotherapy (HIPEC) is delivered under hyperthermic conditions and used to increase chemotherapy penetration into peritoneal tumors, including advanced EOC [12]. Randomized trials, meta-analyses, and retrospective studies have all shown that HIPEC is feasible and associated with prolonged ovarian cancer survival rates [13–15]. Some studies have demonstrated that despite the promise of HIPEC, HIPEC did not improve patient survival data compared with therapy without HIPEC [3, 14, 16]. Interestingly, in a meta-analysis of patients with primary EOC, Huo et al. showed that HIPEC combined with CRS was associated with a significantly improved 2-, 3-, 4-, 5-, and 8-year OS compared with CRS alone [17].

In those studies, different drug and dose ranges of HIPEC were used, which resulted in dissimilar survival times. In some studies, HIPEC was administered only after optimal cytoreductive surgery using cisplatin or a combination of doxorubicin, paclitaxel, or mitomycin C [8, 18–20]. In other studies, patients received HIPEC with different doses of cisplatin [14, 21–23]. Numerous investigations conducted to assess dose dense chemotherapy have shown clinical benefits. For instance, Ba M, et al. showed that HIPEC procedures with three sessions were performed on the first, third, and fifth days after CRS was comparable to other HIPEC procedures [22]. Therefore, we referenced the procedure that have three sessions and defined them as Dense HIPEC with cisplatin.

In this single-center study, we sought to retrospectively analyze the role of IDS combined with dense HIPEC with cisplatin in patients with primary FIGO stage III serous EOC after NACT compared with IDS alone.

## Methods

### Patients

This retrospective study was approved by the Human Ethics Committee of Henan Provincial People’s Hospital in China, and written informed consent was obtained from each patient prior to all treatment procedures. All patients in this study were diagnosed with FIGO stage III high-grade serous ovarian cancer, which based on surgical findings and histopathological results, between January 1, 2012 and April 31, 2017 at the Department of Gynecology, Henan Provincial People’s Hospital. Staging was done based on the 1988 International FIGO staging system between January 1, 2012 and December 31, 2012, the 2013 International FIGO staging system between January 1, 2013 and December 31, 2013, and the 2014 International FIGO staging system between January 1, 2014 and April 31, 2017 [24]. The medical records of all patients were reviewed, and all patients were followed up until March 31, 2020.

All patients who demonstrated an Eastern Cooperative Oncology Group (ECOG) score 0–1 [25, 26] in our study underwent 2–4 cycles of NACT with a combination of carboplatin (area under the curve of 5–6 mg per milliliter per minute, AUC 5–6) and paclitaxel (175 mg per square meter of body-surface area, 175 mg/m^2^). After NACT, IDS was performed using peritonectomy. Complete cytoreductive surgery was defined as surgery that resulted in no visible disease (residual disease classification, R0), optimal cytoreductive surgery as surgery that resulted in the presence of one or more residual tumors measuring ≤1 cm in diameter (R1), and incomplete cytoreductive surgery as surgery that resulted in the presence of one or more residual lesions measuring > 1 cm in diameter (R2) [27]. In this study, regardless of whether satisfactory tumor cell reduction is achieved after IDS (single or more residual lesions ≤1 cm), HIPEC was performed with the consent of patients and family members. According to the patient’s request and a multidisciplinary discussion among general surgeons, gynecologists, and physicians, patients were divided into two groups: an IDS plus dense HIPEC group and an IDS alone control group.

### Inclusion and exclusion criteria

The inclusion criteria were as follows. (1) Patients aged ≥18 years. (2) Patients that underwent pretreatment and preoperative evaluation with computed tomography (CT) or magnetic resonance imaging (MRI), pelvic ultrasound, and had tumor markers (including cancer antigen 125 [CA125] and human epididymis protein 4 [HE4]) as well as routine blood tests. (3) A pathologic diagnosis confirmed by ascites cytology or biopsy pathology. (4) Patients diagnosed with FIGO stage III serous EOC (high-grade serous ovarian cancer). (5) Patients with normal blood counts, and adequate renal function. (6) Patients with an ECOG performance status of 0–1.

The exclusion criteria were as follows. (1) Recurrent peritoneal ovarian cancer patients. (2) Patients with previous primary cytoreductive surgery. (3) Patients with the presence of extra-abdominal metastasis. (4) Patients that treated with bevacizumab or poly ADP-ribose polymerase inhibitors. (5) Patients that with poor general health, renal insufficiency, heart failure, or the existence of a lesion in the central nervous system.

### IDS plus dense HIPEC group

After the IDS procedure, dense HIPEC was administered using a custom-developed high-precision body cavity hyperthermic perfusion treatment system (BR-TRG-II, Bright Medical Technology Co., Ltd., Guangzhou, China) through closed procedures [22]. All patients underwent three sessions of HIPEC, and each session was performed in the general ward on the first, third, and fifth days after surgery with 60 min of cisplatin (60 mg/m^2^).

In brief, after cytoreductive surgery, four perfusion chemotherapy catheters with multiple side holes were placed intra-abdominally; one was placed beneath each hemidiaphragm and two were placed in the pelvis. The abdomen was filled with saline that circulated continuously using the HIPEC device. By circulation of the heated saline, an intra-abdominal temperature of 42–43 °C was maintained. Cisplatin (60 mg/m^2^) was perfused in equal amounts during each 60 min HIPEC session with a 450–600 mL/min flow rate. The total perfusion volume was 3500–4500 mL, which was adjusted such that the entire abdomen was exposed to the perfusate. Four abdominal drains were closed during the intraperitoneal intraoperative chemotherapy and opened after 24 h, and all the perfusion liquid was drained prior to starting the next session. Four catheters were removed 2 days after the final HIPEC session. During HIPEC performed in the general ward, patients were attended by a dedicated therapist and monitored with electrocardiograph (ECG) monitoring. Meanwhile, the intervention treatment comprising dezocine and diazepam was administered to the patients. All patients received additional 3–4 cycles of systemic chemotherapy (paclitaxel 175 mg/m^2^ plus carboplatin AUC 5–6) 2 weeks after the final HIPEC treatment. Before every HIPEC session, bone marrow, renal and hepatic function, parameters of blood electrolytes, and blood coagulation were examined.

### IDS alone control group

We retrospectively selected FIGO III serous ovarian cancer patients who underwent 2–4 cycles of NACT (paclitaxel 175 mg/m^2^ plus carboplatin AUC 5–6) followed by IDS. After surgery, 3–4 cycles of intravenous paclitaxel (175 mg/m^2^) and carboplatin (AUC 5–6) were administered every 21 days.

### Study variables

The following information was reviewed and collected. Patient demographics, medical history, tumor markers, pathologic characteristics, chemotherapy regime including the dose, surgical procedures, tumor debulking status according to the visible residual disease classification [28], the pre- and post-HIPEC lab values (absolute neutrophil count, platelets, hematocrit, and hemoglobin), and HIPEC techniques and drugs, patient adverse events, length of hospital stay, and the cost of hospital data.

### Follow-up

During follow-up after the completion of chemotherapy, physical examinations and measurements of CA125 and HE4 levels were performed every 3 months for 2 years and then every 6 months until the five-year point. CT or MRI was performed at least 1, 6, 12, and 24 months after the last cycle of chemotherapy.

The co-primary endpoints of this study were the survival curves for progression-free survival (PFS) and OS. Clinical, radiologic, and CA125 progression after the initial induction chemotherapy and time to normalization were identified as variables when assessing PFS. OS was defined as the time from the date of initiation of NACT to the date of death from any cause or final follow-up. Safety assessments were performed according to the US National Cancer Institute’s Patient-Reported Outcomes version of the Common Terminology Criteria for adverse events (PRO-CTCAE) [29].

### Statistical analyses

All statistical analyses were performed using SPSS statistical software (version 24.0 for Windows, IBM Corp., Armonk, NY, USA). The initial data analysis was conducted with a descriptive statistical approach. We used the Mann-Whitney U test for continuous variables and the χ^2^ test for categorical variables. Survival analysis was performed using the Kaplan-Meier approach and the median follow-up was calculated using the reverse Kaplan-Meier approach. Hazard ratios (HRs) were estimated using a Cox proportional hazard model with a 95% Wald confidence interval (95% CI). All *p* values were two-sided with the level of significance set at *p* < 0.05.

## Results

### Patient demographics and clinical characteristics

A total of 197 FIGO stage III serous ovarian cancer patients (high-grade serous ovarian cancer) were reviewed, of whom 121 underwent IDS plus dense HIPEC (IDS plus dense HIPEC group) and 76 underwent IDS (IDS alone control group). There were no differences between the two groups in terms of patient age (*p* = 0.337) or body mass index (BMI; *p* = 0.277). Optimal cytoreductive surgery (R0 + R1) was achieved in 113 (93.4%) patients in the IDS plus dense HIPEC group and 70 (92.1%) patients in the IDS alone control group (*p* = 0.734). Although there were no differences in patient age (*p* = 0.337), the median duration of hospitalization after surgery was 8.91 (SD = 1.89) days in the IDS plus dense HIPEC group, which was longer than 8.07 (SD = 1.80) days in the IDS alone group (*p* = 0.001). In the IDS plus dense HIPEC group, patients were treated with a mean of 2.95 (SD = 0.62) cycles of NACT compared with 2.86 (SD = 0.60) cycles in the IDS alone group (*p* = 0.442). The individual surgery groups’ demographic and clinical characteristics are described in Table 1.

**Table 1:** Demographic and clinical characteristics of patient between groups

Variable	IDS + HIPEC (*n* = 121)	IDS alone (*n* = 76)	*p*-value
Demographic mean (SD)			
Age	58.38 (10.89)	55.20 (10.87)	0.337ª
BMI (kg/m^2^) (SD)	26.77 (2.78)	26.65 (2.40)	0.277ª
Neo-adjuvant chemotherapy cycles, n (%)			0.442^b^
Two cycles	28 (23.1)	20 (26.3)	
Three cycles	74 (61.2)	47 (61.8)	
Four cycles	19 (15.7)	9 (11.8)	
Cyto-reductive surgery, n (%)			0.734^b^
Optimal (R0 + R1)	113 (93.4)	70 (92.1)	
Sub-optimal (residual disease > 1 cm)	8 (6.6)	6 (7.9)	
Bowel resection, n (%)			0.523^b^
No bowel resection performed	101 (83.5)	66 (86.8)	
Bowel resection performed	20 (16.5)	10 (13.2)	
Mean duration of hospitalization-days (SD) after surgery	8.91 (1.89)	8.07 (1.80)	0.001^a^
Median time to postoperative systemic treatment with chemotherapy (SD)	22 (2.37)	15 (1.87)	0.001^a^
Performance status (ECOG), n (%)			0.980^b^
0	38 (31.4)	24 (31.6)	
1	83 (68.6)	52 (68.4)	
TC chemotherapy cycles after IDS (%)			0.015^b^
3 cycles	6 (5.0)	5 (6.6)	
4 cycles	58 (47.9)	49 (64.5)	
5 cycles	57 (47.1)	22 (28.9)	
Total of TC cycles (SD)	6.73 (0.53)	6.48 (0.66)	0.047^b^

^a^Analysed using an Independent-Samples t-test; ^b^Analysed using the Mann-Whitney U test. ECOG: Eastern collaborative oncology group. TC: Paclitaxel and carboplatin

### PFS and OS

**Fig. 1:**
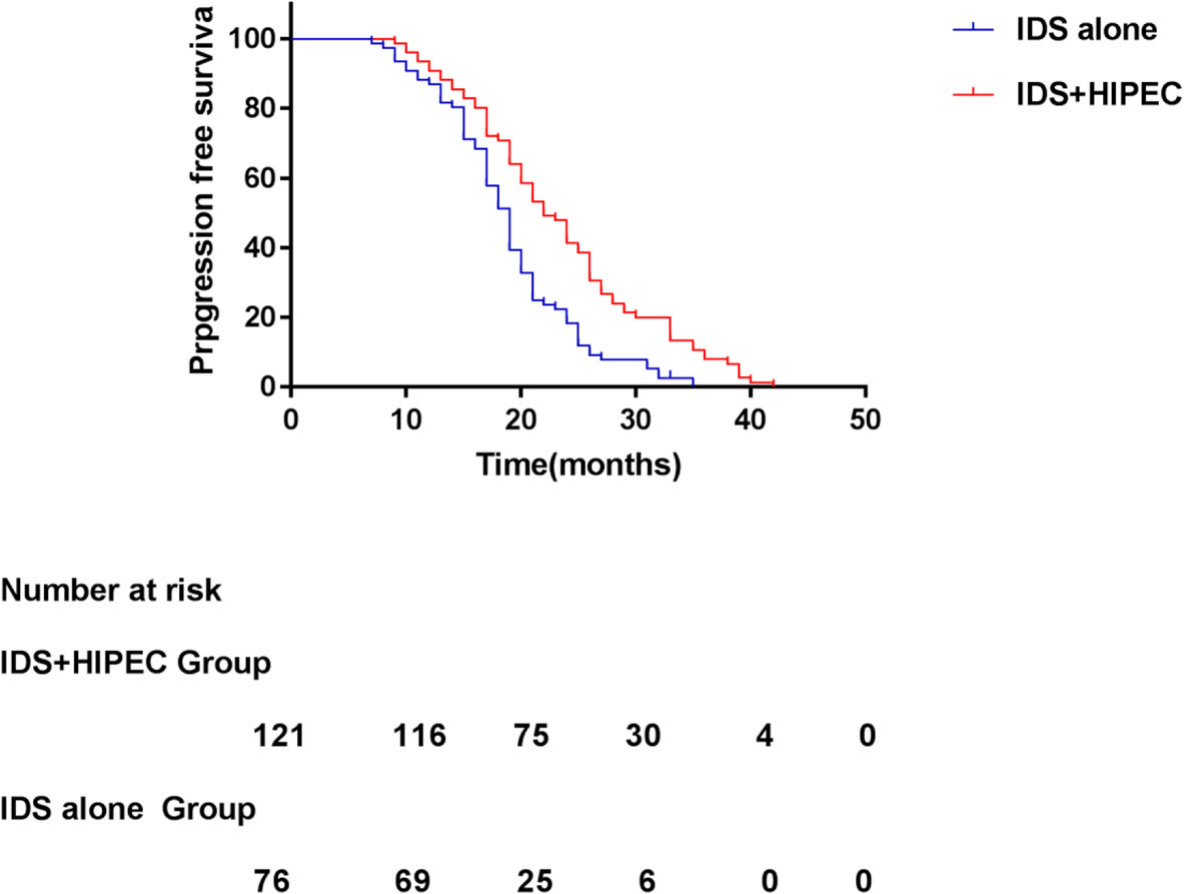
Prpgression free survival for the ovarian cancer patients who underwent IDS with or without HIPEC with cisplain

**Fig. 2:**
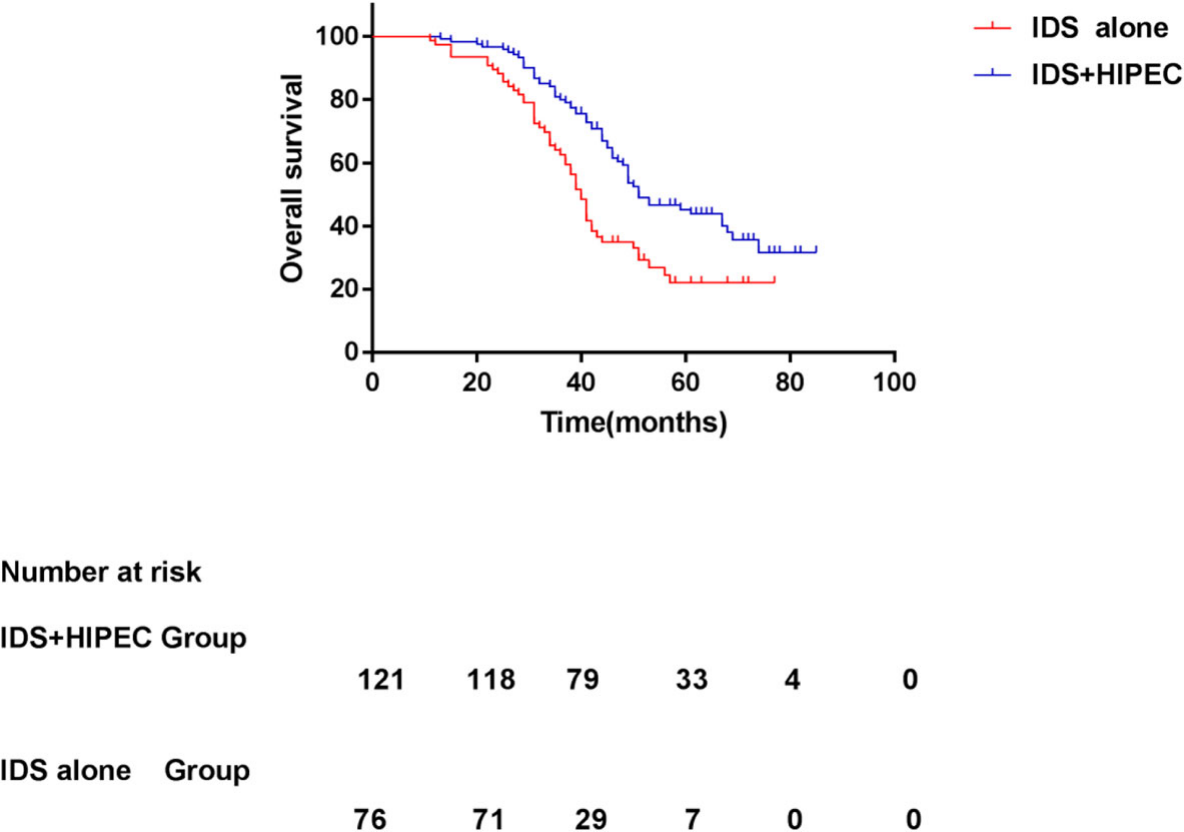
Overall survival for the patients who underwent IDS with or without HIPEC with cisplatin

The median follow-up was 46 and 38 months in the IDS plus dense HIPEC and IDS alone group, respectively. In the IDS plus dense HIPEC group, the median PFS was 24 (95% CI: 21.38–26.62) months compared with 19 (95% CI: 17.81–20.19) months in the IDS alone group (*p* = 0.000), suggesting that the IDS plus dense HIPEC group experienced a significantly longer PFS than the IDS alone group (Fig. 1). Of the 197 patients, 142 patients (72.1% of the recurrences) were detected based on imaging and 55 patients (27.9%) were detected based on an increase in CA125 levels alone (more than 35 U/mL). Overall, 69 of the 121 patients (52.02%) in the IDS plus dense HIPEC group and 55 of the 76 patients (72.37%) in the IDS alone group died during the follow-up period. The median OS was 51 (95% CI: 42.73–59.27) months in the IDS plus dense HIPEC group and 40 (95% CI: 37.59–42.41) months in the IDS group (*p* = 0.001, Fig. 2). Significant differences were observed between the groups in both the median PFS (HR 0.46, 95% CI: 0.33–0.65, *p* = 0.000) and median OS (HR 0.52, 95% CI: 0.35–0.78, *p* = 0.001).

### Adverse events due to HIPEC

Patients treated with IDS plus dense HIPEC received a mean of 4.4 (SD = 0.59) cycles of carboplatin and paclitaxel chemotherapy after a median duration of 22 days of postoperative systemic treatment with chemotherapy. Patients treated with IDS alone underwent a mean of 4.2 (SD = 0.56) cycles after a median duration of 15 days of postoperative systemic treatment with chemotherapy (*p* = 0.000). In the IDS plus HIPEC group, a total of seven patients could not complete three sessions of HIPEC, implying they only underwent two sessions of HIPEC. Two patients developed fever and had a body temperature as high as 39 °C for 3 days, and four patients had intestinal obstruction symptoms after hyperthermic perfusion on both sides. In one patient, the third session of HIPEC was terminated because of intestinal anastomosis bleeding.

In the IDS plus HIPEC group, no adverse events were recorded in 55 (45.5%) of the 121 patients in the group, and 23 (19.0%) of the 121 patients were reported to have grade III or IV adverse events. Conversely, in the IDS alone group, no adverse events were recorded for 42 (55.3%) of the 76 patients, and 14 (18.4%) were reported to have grade III-IV adverse events. The main adverse events observed in this study are summarized in Table 2. The most common grade I-II adverse event observed in this study was an electrolyte disturbance, and the most common grade III-IV adverse event was ileus. All HIPEC procedures were performed in the general ward and not in the intensive care unit (ICU). No deaths occurred within the first 30 days postoperatively in the two groups of patients and neither did severe fatal complications in the IDS plus dense HIPEC group.

**Table 2:** Adverse events for the ovarian cancer patients treated with IDS plus HIPEC (*n* = 121) and IDS alone (*n* = 76)

Adverse events n (%)	Grade I or II		Grade III or IV	
	IDS + HIPEC	IDS alone	IDS + HIPEC	IDS alone
Haematology				
Leucocytopenia	21 (17.4)	2 (2.6)	0	0
Thrombocytopenia	9 (7.4)	0	0	0
Anemia	30 (24.8)	18 (23.7)	1 (0.8)	2 (2.6)
Electrolyte disturbance*	38 (31.4)	20 (26.3)	4 (3.3)	2 (2.6)
Thromboembolism	10 (8.3)	5 (6.6)	3 (2.5)	2 (2.6)
Nausea	27 (22.3)	15 (19.7)	0	0
Emesis	16 (13.2)	9 (11.8)	0	0
Nephrotoxicity	5 (4.1)	0	0	0
Abdominal pain	26 (21.5)	13 (17.1)	2 (1.7)	0
Ileus	6 (5.0)	2 (2.6)	10 (8.3)	5 (6.6)
Anastomotic hemorrhage	4 (3.3)	3 (3.9)	2 (1.7)	0
Infection	15 (12.4)	11 (14.5)	3 (2.5)	3 (3.9)

Electrolyte disturbances included hyponatremia, hypochloridemia, hypokalemia, hypocalcemia, hypomagnesemia and hypophosphatemia. Infection included lung infection, urinary tract infection, urethral infection, stoma site infection, sepsis, bacteremia, abdominal infection

## Discussion

In most cases of advanced ovarian cancer, peritoneal metastasis is the primary site of spread and cause of treatment failure [9]. Several randomized clinical trials have shown that postoperative intraperitoneal and intravenous chemotherapy improved survival in patients with optimally resected stage IIIC ovarian cancer compared with intravenous chemotherapy alone [26, 30–32]. Although the survival benefits of intraperitoneal chemotherapy have been reported in a previous Gynecologic Oncology Group study [31], the toxicity and significantly high incidence of side effects have prevented this treatment from being widely adopted in clinical practice [12, 26]. In recent years, the application of HIPEC to ovarian cancer has garnered interest because of its advantages over standard intraperitoneal chemotherapy [12].

Nevertheless, the therapeutic of HIPEC in ovarian cancer has been controversial. Some studies have demonstrated that HIPEC does not improve OS compared with therapy without HIPEC [13]. However, an increasing number of studies have demonstrated that the addition of HIPEC significantly improves the prognosis of ovarian cancer [1, 22, 33–35], especially in randomized controlled trials [14, 16, 36]. The drug and dose range of HIPEC are important considerations that can affect survival times. In some studies, HIPEC was performed only after optimal cytoreductive surgery, either as an open procedure or a closed system, which used cisplatin or a combination of doxorubicin, paclitaxel, or mitomycin C [8, 18–20]. In other studies, patients underwent HIPEC with different doses of cisplatin [14, 22, 23]. In the study by Antonio et al., patients were treated with HIPEC and paclitaxel alone [21]. Hence, several investigations have shown that dose dense chemotherapy provides clinical benefits. Therefore, this study focused on the feasibility of dense HIPEC, which involves increasing the rate of chemotherapy with three sessions of HIPEC, in FIGO stage III serous EOC.

In the present study, the median PFS was 24 months in the IDS plus dense HIPEC group, which was significantly better than the 19 months observed in the IDS alone control group, suggesting the feasibility of dense HIPEC in patients with FIGO stage III serous EOC. Two groups of patients in this study received similar preoperative treatment and had the same disease stage and pathological type. No significant differences in cytoreductive surgery, pathological grade, or the total number of cycles of systematic intravenous chemotherapy existed between the two groups. Mendivil et al. reported improved PFS with HIPEC compared with intravenous chemotherapy (25.1 months versus 20.0 months, respectively; *p* = 0.024) [13], which corroborates the results of this study. Similarly, a randomized, controlled, open-label trial also documented an increased PFS (14.2 months versus 10.7 months) in cohorts treated with HIPEC chemotherapy [14].

Although previous studies reported favorable PFS results, OS benefits have conventionally been considered as the most dependable endpoint in assessing cancer-related treatments. In a multicenter, open-label, phase 3 trial [14] 245 patients who received NACT were randomly assigned to undergo interval cytoreductive surgery either with or without HIPEC with cisplatin (100 mg/m^2^). A significant improvement in OS was seen: 45.7 months versus 33.9 months in the surgery plus HIPEC and surgery alone group, respectively. Notably, our results showing a median OS of 51.0 months for the IDS plus dense HIPEC group versus 40.0 months for the IDS alone group. Nevertheless, a recent retrospective case-control study of 56 patients treated for primary advanced ovarian cancer who underwent interval surgery with or without HIPEC demonstrated that although OS was better in the case group than in the control group (*p* = 0.048), PFS was not significantly different (13.2 months versus 13.9 months, *p* = 0.454) [8]. Conversely, another retrospective case-control study showed that PFS was significantly longer with HIPEC than without, but no OS advantages were identified. The differences in these results may have several explanations. First, follow-up differed in the two studies, and a shorter duration was inherent to the HIPEC group [13]. Second, the studies were retrospectively evaluated, and selection bias may have influenced the outcomes [13]. Third, a relatively small number of patients were included to obtain the results. Last, the rate of the peritoneal metastasis differed, which is a critical determinant of survival in advanced ovarian cancer [8].

Unsurprisingly, we observed a reasonable toxicity profile in the patients receiving dense HIPEC. No grade IV adverse events were recorded in any of the 121 patients, and no adverse events were recorded in 55 (45.5%) patients, however, 43 patients developed grade II nausea during the three sessions of HIPEC. Notably, we did not observe any grade III-IV leukocytopenia, thrombocytopenia, or renal failure. Similarly, several retrospective studies did not report any evidence of grade III-IV toxicity in their experience of treating advanced ovarian cancer patients with HIPEC [13, 37].

To the best of our knowledge, HIPEC as a single treatment has only been administered at the end of cytoreductive surgery in the operating room or the ICU [8, 13, 14, 22], thus increasing not only patient safety and comfort but also medical costs [38]. However, in our center, dense HIPEC is delivered with cisplatin after cytoreductive surgery in the general ward, and no adverse events were found to be associated with this. Furthermore, the patient tolerability was excellent. The median time of postoperative systemic treatment with chemotherapy was 22 days, which was significantly lower than the 33–49 days reported in previous studies [8, 14].

In addition, specific pathologic subtypes affect the prognosis of advanced ovarian cancer. To reduce the impact of pathological type and disease stage on survival in the present study, we only included FIGO stage III high-grade serous EOC patients. Several limitations should be considered in this study. First, the retrospective nature of our analysis reduced our ability to draw reliable conclusions. Second, although NACT is increasingly used as the primary treatment for advanced ovarian cancer [39], patients who undergo primary debulking surgery have a significant survival benefit compared with those who undergo NACT. Third, this study showed that significant benefits in OS and PFS were associated with dense HIPEC, however, multivariate analyses were not performed to elucidate the risk factors of prognosis nor evaluate the impact of optimal cytoreductive surgery, CA125 level, age, and other variables. In a retrospective multicenter study of 78 patients by Le Saux, et al., the univariate analysis demonstrated that age ≥ 50 years, peritoneal cancer index (PCI) ≤8, and CA125 levels < 100 were significantly associated with long-term survival in patients with EOC following cytoreductive surgery and HIPEC [40]. However, our data were limited to optimal or suboptimal cytoreductive surgery [27], and it may have been preferable to further stratify the surgery categories into complete (R0), optimal < 1 cm (R1), and suboptimal (R2) groups [31].

At present, there are many ongoing randomized Phase 2 and 3 trials involving primary and recurrent disease as well as patients receiving NACT [41]. Many of these trials intend to assess the impact of adding HIPEC to cytoreductive surgery on PFS and OS [41] as well as on morbidity, quality of life, and pharmacokinetics [42]. However, to the best of our knowledge, few randomized controlled clinical trials of dense HIPEC training and administration with the agents and protocols used in our study are ongoing.

## Conclusions

This retrospective study of FIGO stage III serous EOC patients indicates that IDS plus dense HIPEC results in longer survival than IDS alone, and this treatment is reasonably well tolerated by patients. Future trials investigating the efficacy and safety of dense HIPEC plus debulking surgery in a similar setting are required.

## Data Availability

The datasets used or analyzed during the current study are available from the corresponding author on reasonable request.

## Abbreviations

IDS: Interval debulking surgery
HIPEC: Hyperthermic intraperitoneal chemotherapy
EOC: Epithelial ovarian cancer
PFS: Progression-free survival
OS: Overall survival
HR: Hazard ratio
CI: Confidence interval
CRS: Cytoreductive surgery
NACT: Neoadjuvant chemotherapy
ECOG: Eastern cooperative oncology group
CT: Computed tomography
MRI: Magnetic resonance imaging
CA125: Cancer antigen 125
HE4: Human epididymis protein 4
ECG: Electrocardiograph
BMI: Body mass index
ICU: Intensive care unit
PCI: Peritoneal cancer index
TC: Paclitaxel and carboplatin
